# Impaired Preoperative Spinopelvic Function Does Not Compromise the Patient-Reported Outcome at 2-Years After Total Hip Arthroplasty

**DOI:** 10.1016/j.artd.2026.102148

**Published:** 2026-09-24

**Authors:** Henryk Haffer, Maximilian Muellner, Luis Becker, Abelina Schneider, Tom Folkerts, Sebastian Hardt, Carsten Perka, Matthias Pumberger

**Affiliations:** Center for Musculoskeletal Surgery, Charité - Universitätsmedizin Berlin, Corporate Member of Freie Universität Berlin, Humboldt-Universität zu Berlin, and Berlin Institute of Health, Berlin, Germany

**Keywords:** Spinopelvic mobility, Hip replacement, Patient-reported outcome, Spinopelvic stiffness, Hip dislocation

## Abstract

**Background:**

Impaired spinopelvic function contributes to instability after total hip arthroplasty (THA). It remains unclear whether preoperative spinopelvic function affects patient-reported-outcomes (PROs) in patients undergoing THA. We hypothesized that preoperative pathological spinopelvic function is associated with worse postoperative PROs at 2-years-follow-up (2Y-FU) after THA.

**Methods:**

A follow-up study of a prospective cohort undergoing primary THA was performed. PROs at 2Y-FU included Hip-Disability-and-Osteoarthritis-Outcome-Score (HOOS), Harris-Hip-Score (HHS), and Subjective-Hip-Value (SHV). Pelvic mobility (∆Pelvic Tilt = PT_standing_-PT_sitting_) was defined as a key parameter. Pelvic mobility was classified as stiff(∆PT<10°), normal(∆PT≥10°-30°) and hypermobile(∆PT>30°). PROs were compared between groups stratified according to pelvic mobility. The relationship between spinopelvic function, cup position and PROs at 2Y-FU was analyzed.

**Results:**

A total of 144 patients (N = 79 females; mean 68.9years; mean body mass index 26.9kg/m^2^, follow-up rate 73.1% (144/197)) were followed-up (mean 29.1months). HOOS-subdomains, HHS and SHV did not differ between the groups stratified for preoperative pelvic mobility at 2Y-FU. Higher pelvic mobility and lower standing and sitting lumbar lordosis were associated with better HOOS and HHS. No dislocation was reported in our cohort.

**Conclusions:**

This study demonstrated that impaired preoperative pelvic mobility does not adversely affect PROs in patients undergoing elective THA at 2Y-FU. It can be concluded that primary THA patients without risk factors (eg, spinal fusion, neurological disease) do not face limited outcomes, regardless of their preoperative pelvic mobility. Nevertheless, spinopelvic function and functional cup position appear to be associated with PROs, and the underlying mechanisms require further investigation.

## Introduction

Outcomes after primary total hip arthroplasty (THA) are generally favorable; nevertheless, THA dislocation remains a relevant failure mechanism [1]. Spinopelvic function has recently gained increased attention as a potential contributing factor to THA instability [2,3]. It is known that limited mobility of the pelvis and spine due to degeneration or spinal fusion increases the risk for THA dislocations [[4], [5], [6]]. Spinopelvic function is the interaction between hip, pelvis, and spine, enabling different body positions and an erect posture [7]. It is shaped by the complex interaction of the back, abdominal, pelvic, and thigh muscles [8]. The physiological change in pelvic tilt from standing to sitting (pelvic mobility) is considered a main parameter in spinopelvic function [9].

In addition to the established link between the risk of dislocation and pathological spinopelvic function in high-risk cohorts, evidence remains limited and conflicting on whether patient-reported outcomes in regular THA cohorts are influenced by impaired spinopelvic function prior to surgery. Innmann et al. [9] found no influence of preoperative spinopelvic function on preoperative patient-reported outcomes (PROs) in patients undergoing primary THA. Polat et al. [10] demonstrated an association of normal spinopelvic motion (Δsacral slope 11-29°) with improved functional outcomes after THA. Another study found no association of spinopelvic parameters and PROs 3 months after THA [11]. The studies included smaller cohorts (n = 100/ 60/ 90) and a shorter follow-up (FU) period of 12 months. Therefore, the current literature remains inconclusive and larger studies with longer FU are warranted.

We therefore conducted this FU study of a prospective cohort to investigate if the patient reported outcomes at 2 years after THA using validated outcome metrics is influenced by the preoperative spinopelvic function. First, we compared the PROs at 2Y-FU between the groups stratified according to preoperative pelvic mobility. Second, we investigated the relationship between preoperative and postoperative spinopelvic function, functional cup position, and PROs at 2Y-FU.

## Material and methods

### Subjects and study design

A clinical FU study of an established prospective observational study cohort of patients who underwent elective primary unilateral THA in our department between 20^th^ September 2019 and 30^th^ November 2020 was conducted from 1^st^ May 2022 to 1^st^ March 2023 [12]. The investigation was conducted in accordance with the Declaration of Helsinki, has been approved by the institutional ethics board (EA2/021/22) and patients have given their informed written consent. The minimum FU was 22 months after THA and a clinical examination, evaluation of validated PROs, assessment of complications and a radiographic assessment were performed. An overview of the study design is shown in Figure 1.

**Figure 1:**
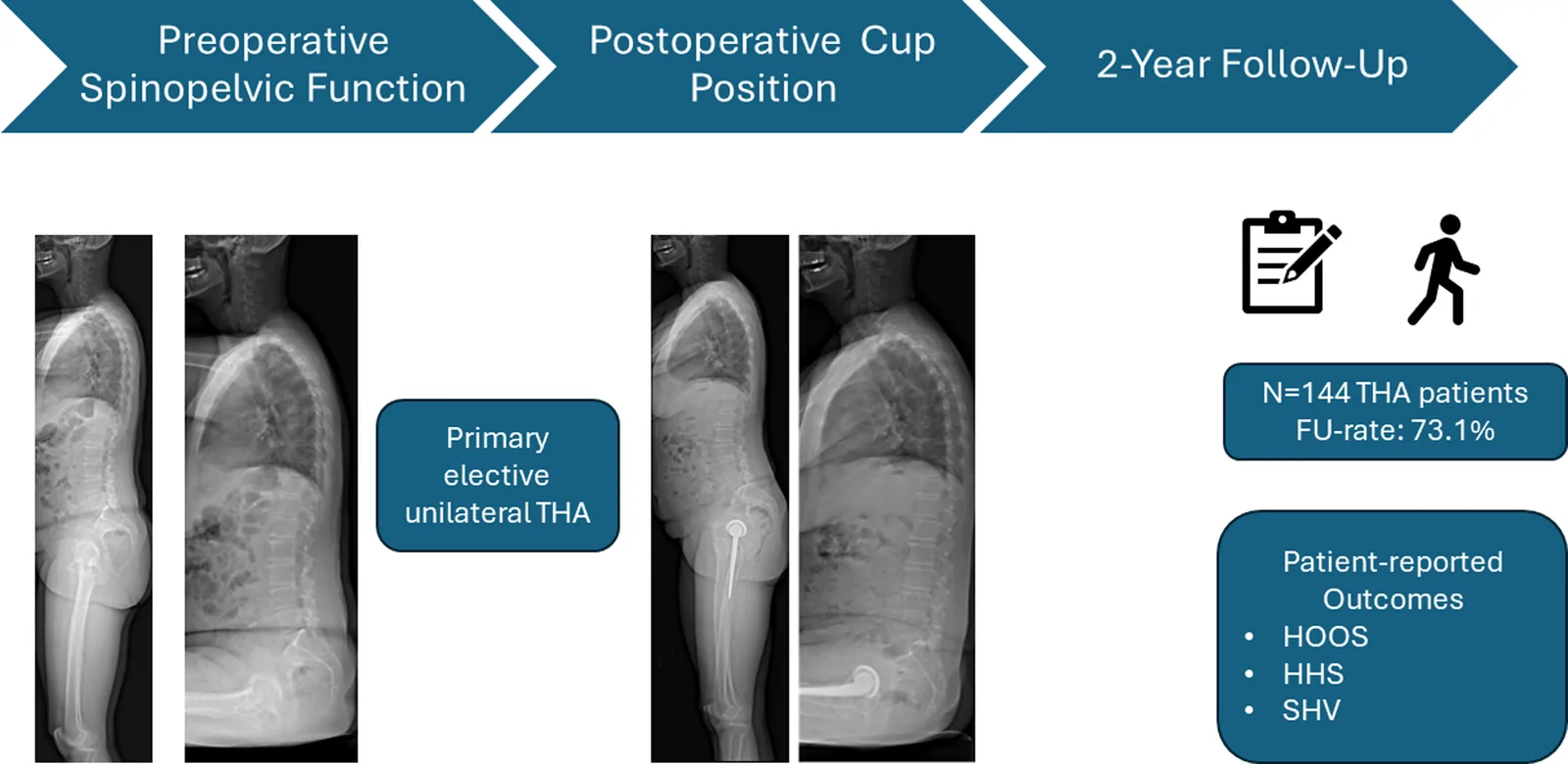
Overview of the study design. FU-rate, follow-up rate; HOOS, Hip Disability and Osteoarthritis Outcome Score; HHS, Harris Hip Score; SHV, Subjective Hip Value.

In the primary patient cohort, the exclusion criteria were any nonelective surgery, bilateral planned THA, severe hip dysplasia with subsequent THA and femur osteotomy, any form of revision THA, ankylosing spondylitis, preexisting spinal fusion at any level, and neurological pre-existing conditions (eg, M. Parkinson) that significantly influence posture. THAs were performed by four board certified surgeons in supine position via an anterolateral approach and using the Lewinnek safe zone as the target for the implant positioning [13]. All patients were treated with cementless cups, UHMWPE or ceramic-inlays, ceramic heads (32 and 36 mm), and cementless stems (all implants ZimmerBiomet, Warsaw, IN or Smith & Nephew, London, UK) and our standardized rehabilitation protocol.

### Patient-reported outcome measures

Hip Disability and Osteoarthritis Outcome Score (HOOS) is one of the established outcome tools assessing the patient's hip-related health using a 40-item questionnaire. Each item has five possible responses. Standardized answer options are given in 5 Likert boxes with scores from 0 to 4 (no, mild, moderate, severe, and extreme) to answer the questions. The HOOS has five subcategories: pain, symptoms and stiffness, activities of daily living, function in sports and recreational activities, and quality of life.

### Harris Hip Score

The Harris Hip Score (HHS) is a specific, validated instrument for the assessment of complaints of the hip joint. It is a clinician and PRO measure and as such is recorded partly by the patient and partly by a qualified healthcare professional. With ten items, the HHS covers four domains and is used for many questions relating to diseases of the hip joint: pain, function, absence of deformities, and range of motion. The HHS can have a maximum value of 100 points and utilizes this scale to indicate the function of the joint: The lower the score, the worse the function of the hip joint.

### Subjective Hip Value

The Subjective Hip Value (SHV) is a valid, reliable, and responsive instrument for assessing hip function in primary THA patients [14]. It is a single-item value, assessing hip function with 1 question: "What is the overall percent value of your hip if a completely normal hip represents 100%?"

### Complications

The FU study collected all severe complications such as periprosthetic infection, loosening, instability/dislocation, periprosthetic fracture, and if revision surgery was performed. Charlson Comorbidity Index was acquired to estimate concurrent comorbidities.

### Radiographic analysis

The patients received preoperatively and postoperatively after 1 week and at the 2Y-FU a radiographic examination including the lumbar spine, the pelvis and the femur from lateral and anterior–posterior in standing and sitting position (sitting only at the preoperative and 1 week postoperative examination) using biplanar low-dose stereoradiography (EOS, Paris, France).

Patients were instructed to stand naturally in the standing position, look forward, and place their hands on a support with relaxed upper limbs. In the seated position, they were instructed to sit on a height-adjustable stool with the femur parallel to the floor. The radiological measurements were performed by an experienced orthopaedic surgeon using Merlin Diagnostic Workcenter (Phoenix PACS, Freiburg, Germany) and a randomly selected 25% dataset was measured by a second independent orthopaedic surgeon.

The following parameters were measured (Fig. 2 and Supplement Table 1 for definition): lumbar lordosis (LL), pelvic tilt (PT), pelvic femoral angle. The difference between the standing and sitting values was designated as delta (Δ). To classify pelvic mobility, we determined Δ PT, preoperatively and postoperatively. ΔPT < 10° were classified as stiff, ≥10-30° as normal and >30° as hypermobile [9]. The measurement of cup anteversion and inclination were conducted in standing and sitting anterior–posterior radiographs using a reliable method [15,16]. Inclination was defined as the angle between the line of the long axis of the ellipse and the interteardrop line and anteversion was defined by the trigonometric equation arc sine (short axis/long axis). The functional cup position is defined as the difference between the standing and sitting anteversion and inclination values, and represents dynamic cup behavior during functional movement.

**Figure 2:**
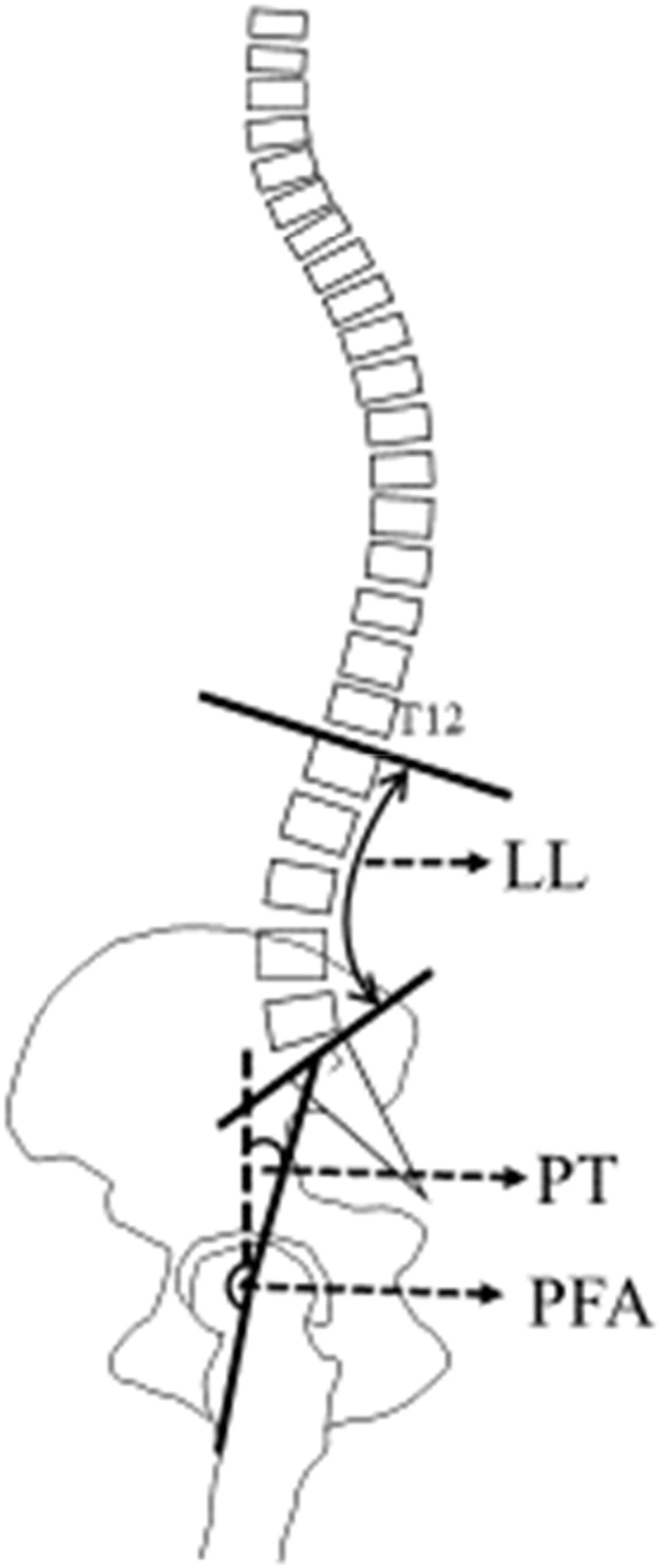
Illustration of radiological spinopelvic parameters. PFA, pelvic femoral angle.

The reasons patients were lost to FU are depicted in Figure 3.

**Figure 3:**
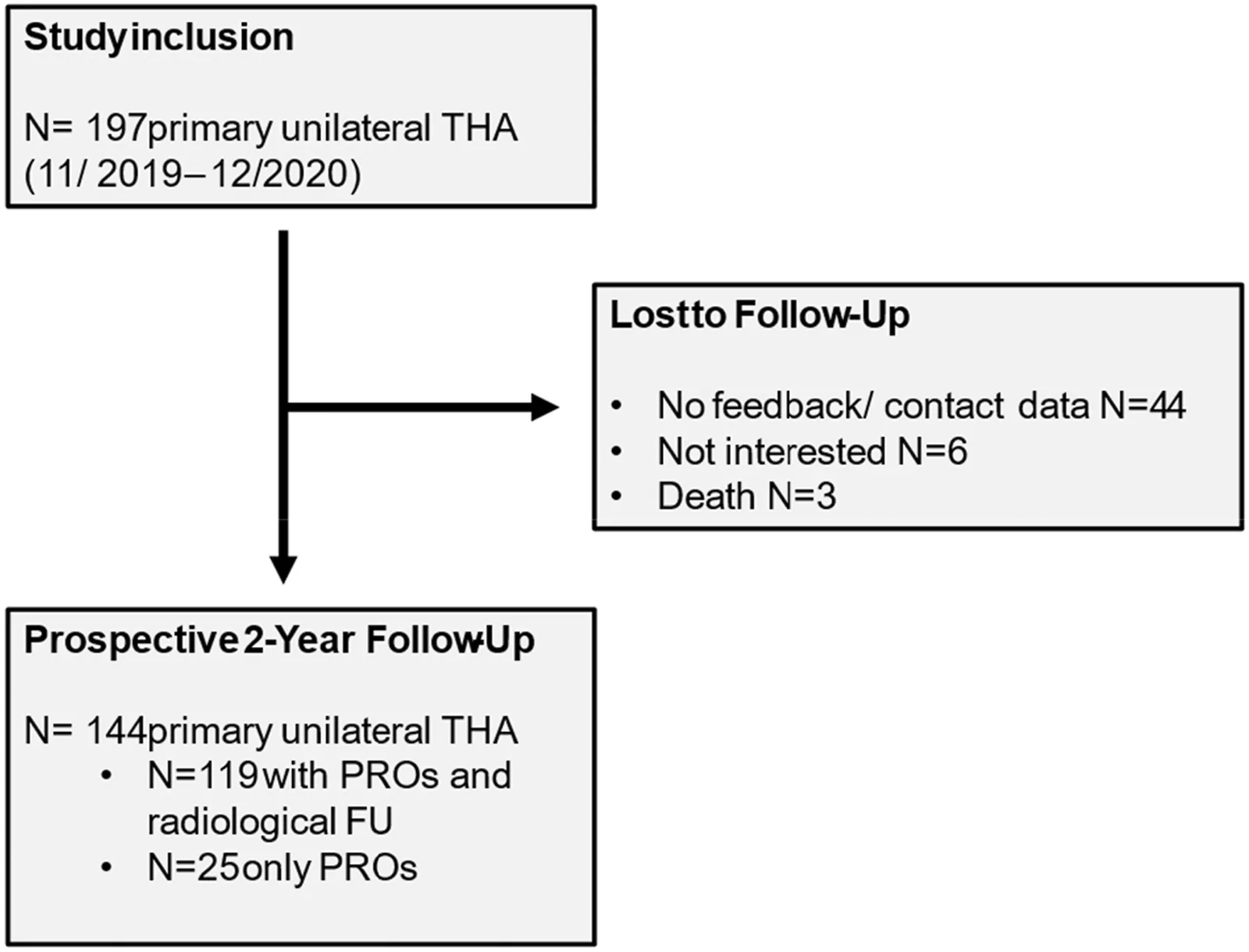
Flowchart of patient inclusion and patients lost to follow-up.

### Data analyses

Differences in HOOS domains among the preoperative pelvic mobility groups were defined as the primary outcome. Differences HHS and SHV among the pelvic mobility groups were defined as secondary outcomes. In addition, the associations between preoperative and postoperative spinopelvic function, functional cup position, and PROs were evaluated as secondary outcomes. Normal distribution was tested with Shapiro–Wilk test. Nonparametric Kruskal–Wallis test and in case of group differences a post hoc analysis by Dunn's test with Bonferroni correction was performed to investigate PRO differences at 2Y-FU between the groups stratified for preoperative pelvic mobility (Δ PT). Linear regression analysis (Spearman’s rank correlation coefficient) was applied to investigate the relationship between spinopelvic mobility parameters, functional cup position and PROs at 2Y-FU. An a priori power analysis was conducted for sample size estimation, based on data from a previously published study [17]. The effect size in their study was 0.35, considered to be small to medium using Cohen's criteria. An a priori sample size calculation indicated that a minimum of 111 patients with FU was required to detect a 20-point difference in the HOOS activities of daily living domain among the three pelvic mobility groups, assuming an α-level of 0.05, a power of 0.80, and an effect size (f) of 0.30. The calculation was performed using GPower version 3.1.9.7 (Heinrich Heine University Düsseldorf, Germany) [18]. Statistical analyses were performed using SPSS Version 27 (IBM Corporation, New York, USA). A significance level of *P* < .05 was assumed for all tests.

## Results

A total of 144 THA patients were followed up (FU rate 73.1% (144/197) and a mean FU of 29.1 months (range 22.1-37.9 month). The patient cohort consisted of 79 female and 65 male patients with a mean age of 68.9 years (standard deviation [SD] 10.8, range 39-89 years), with a mean body mass index of 26.9 kg/m^2^ (SD ± 4.6, range 18.0-47.2 kg/m^2^). The Charlson Comorbidity Index was at mean 3.2 (SD ± 1.7; range 0-7). Two complications were recorded during the FU period, two of which were periprosthetic infections requiring revision. No dislocations were reported.

### Comparison of preoperative pelvic mobility and PROs at 2Y-FU

No differences in age and body mass index were observed between pelvic mobility groups. There is no significant difference between the stratified groups regarding PROs. (Table 1).

**Table 1:** Comparison of preoperative pelvic mobility and PROs at 2Y-FU.

Patient and PRO parameter	Stiff pelvic mobility N = 36 [±SD]	Normal pelvic mobility N = 87 [±SD]	Hypermobile pelvic mobility N = 21 [±SD]	*P* value
Age (years)	67.9 [12.8]	69.8 [9.7]	67.9 [12.8]	.853
BMI (kg/m^2^)	26.9 [5.9]	27.0 [4.2]	26.6 [4.1]	.881
HOOS _Symptoms_	80.2 [15.3]	83.1 [15.8]	84.2 [13.3]	.553
HOOS _Pain_	83.9 [16.5]	84.5 [18.4]	84.2 [13.3]	.894
HOOS _ADL_	76.6 [19.7]	81.7 [19.9]	86.9 [14.1]	.182
HOOS _Sports_	65.2 [24.2]	72.3 [26.1]	73.4 [23.1]	.240
HOOS _QoL_	70.8 [22.4]	72.5 [25.0]	78.0 [18.7]	.598
HHS	82.6 [14.5]	83.5 [17.8]	82.5 [17.5]	.587
SHV	91.8 [8.7]	88.5 [14.7]	82.3 [16.2]	.290

Kruskal–Wallis test was used to detect differences between the groups stratified for pelvic mobility. A significance level of 0.05 was determined. All values are depicted as mean ± standard deviation.

HOOS, Hip Disability and Osteoarthritis Outcome Score with the subscores pain (Pain), symptoms and stiffness (Symptoms), activities of daily living (ADL), function in sports and recreational activities (Sports) and quality of life (QoL).

### Correlation of preoperative spinopelvic function and PROs at 2Y-FU

Higher preoperative PT in sitting position is associated with increased HOOS pain (ϱ = 0.182). Lower LL in standing and sitting is associated to increased HOOS domains symptoms (ϱ = −0.220; ϱ = −0.177), pain (ϱ = −0.187), and activities of daily living (ϱ = −0.182; ϱ = −0.206) (Table 2).

**Table 2:** Correlation of preoperative spinopelvic function and PROs at 2Y-FU.

Spinopelvic parameter	HOOS-symptoms	HOOS-pain	HOOS-ADL	HOOS-sports	HOOS-QoL	HHS	SHV
Δ PT	−0.023	−0.001	−0.076	−0.072	0.025	0.002	0.090
PT_Stand_	0.119	0.167	0.030	0.012	0.057	0.054	0.068
PT_Sit_	0.149	0.182^a^	0.123	0.085	0.033	0.025	−0.014
Δ LL	−0.021	0.104	0.068	0.025	0.020	−0.056	−0.094
LL_Stand_	−0.220^a^	−0.134	−0.182^a^	−0.150	−0.137	−0.083	−0.051
LL_Sit_	−0.177^a^	−0.187^a^	−0.206^a^	−0.167	−0.162	−0.042	−0.001
Δ PFA	−0.081	−0.003	−0.052	−0.052	−0.006	−0.048	−0.068
PFA_Stand_	−0.013	0.053	−0.048	−0.041	−0.019	−0.038	0.038
PFA_Sit_	0.115	0.088	0.063	0.061	0.028	0.048	−0.007

Spearman rank correlation coefficient test was used to determine association between preoperative spinopelvic function and PROs at 2Y-FU. Rho correlation coefficient is depicted. Significant values are marked in bold. A significance level of 0.05 was determined.

HOOS, Hip Disability and Osteoarthritis Outcome Score with the subscores pain, symptoms and stiffness, activities of daily living, function in sports and recreational activities and quality of life. PFA, pelvic femoral angle; Delta, difference between standing and sitting values; Stand, Standing values in lateral radiograph; Sit, Sitting values in lateral radiograph.

a A significance level of 0.05 was determined.

### Correlation of postoperative spinopelvic function and PROs at 2Y-FU

Higher postoperative pelvic mobility (Δ PT) and PT in standing are associated with increased HOOS symptoms (ϱ = 0.206) and pain (ϱ = 0.261; ϱ = 0.179) and higher pelvic mobility (ϱ = 0.190) is associated to increased HHS. Lower postoperative LL in standing is associated to increased HOOS symptoms (ϱ = −0.189) (Table 3).

**Table 3:** Correlation of postoperative spinopelvic function (1W-FU) and PROs at 2Y-FU.

Spinopelvic parameter	HOOS-symptoms	HOOS-pain	HOOS-ADL	HOOS-sports	HOOS-QoL	HHS	SHV
Δ PT	0.206^a^	0.261^b^	0.129	0.110	0.151	0.190^a^	0.092
PT_Stand_	0.146	0.179^a^	0.020	0.018	0.042	0.090	0.019
PT_Sit_	−0.067	−0.079	−0.105	−0.087	−0.130	−0.095	−0.008
Δ LL	−0.132	−0.097	−0.013	−0.028	−0.120	−0.113	−0.096
LL_Stand_	−0.189^a^	−0.129	−0.145	−0.123	−0.126	−0.101	−0.050
LL_Sit_	−0.042	−0.045	−0.099	−0.098	−0.044	−0.018	−0.025
Δ PFA	0.081	0.160	0.125	0.096	0.029	0.001	−0.064
PFA_Stand_	0.036	0.068	−0.023	−0.013	−0.052	−0.040	0.006
PFA_Sit_	−0.023	−0.112	−0.107	−0.050	−0.041	−0.014	−0.086

Spearman rank correlation coefficient test was used to determine association between postoperative spinopelvic function and PROs at 2Y-FU. A significance level of 0.05 was determined.

HOOS, Hip Disability and Osteoarthritis Outcome Score with the subscores pain, symptoms and stiffness, activities of daily living, function in sports and recreational activities and quality of life; PFA, pelvic femoral angle; Delta, difference between standing and sitting values; Stand, standing values in lateral radiograph; Sit, sitting values in lateral radiograph; 1W-FU, 1-week follow-up.

a A significance level of 0.05 was determined.

b A significance level of 0.01 was determined.

### Correlation of functional cup position and PROs at 2Y-FU

A higher difference of cup inclination between sitting and standing (Δ cup inclination) is significantly associated with increased HOOS subgroups symptoms, pain, and quality of life (ϱ = 0.223; ϱ = 0.208; ϱ = 0.190) (Table 4).

**Table 4:** Correlation of postoperative functional cup position (1W-FU) and PROs at 2Y-FU.

Functional cup position	HOOS-symptoms	HOOS-pain	HOOS-ADL	HOOS-sports	HOOS-QoL	HHS	SHV
Δ Cup inclination	0.223^a^	0.208^b^	0.135	0.153	0.190^a^	0.162	0.068
Δ Cup anteversion	0.007	0.044	−0.054	0.061	0.001	0.038	−0.012

Spearman rank correlation coefficient test was used to determine association between functional cup position and PROs at 2Y-FU. A significance level of 0.05 was determined.

HOOS, Hip Disability and Osteoarthritis Outcome Score with the subscores pain, symptoms and stiffness, activities of daily living, function in sports and recreational activities and quality of life. Delta cup inclination, difference of acetabular cup inclination between sitting and standing position; Delta cup anteversion, difference of acetabular cup anteversion between sitting and standing position; 1W-FU, 1-week follow-up.

a A significance level of 0.05 was determined.

b A significance level of 0.01 was determined.

## Discussion

This is the first study evaluating the preoperative spinopelvic function and functional cup position with regards to several established PROs in a 2Y-FU after primary THA. The study did not demonstrate significant differences in PROs between groups stratified for pelvic mobility 2 years after THA.

The existing literature examining spinopelvic function and PROs in primary THA cohorts is scarce. Innmann et al. [9] compared preoperative PROs between groups stratified for preoperative pelvic mobility in a primary THA cohort and found similar to our results no difference. The group of Grammatopoulos et al. studied HOOS-Physical function shortform (PS) outcomes in primary THA patients with a 1-year FU with regards to preoperative and postoperative pelvic mobility [17]. They reported no HOOS-PS differences between preoperative pelvic mobility groups, but a significant worse postoperative HOOS-PS outcome in the hypermobile group, which may also be reflected in our findings, with the tendency of worse SHV outcomes in the hypermobile group.

We consider several factors influencing the interplay between spinopelvic function and PROs and subsequently being responsible for the absence of PRO differences. One possible explanation might be the adaption of spinopelvic function after THA. Ryan et al. and Innmann et al. [19,20] demonstrated a normalization of spinopelvic function after hip replacement. Muellner et al. [21,22] reported an increase of lumbar flexibility, while another study found an increase of pelvic mobility in a short-term follow after THA.

A second explanation may be the characteristics of the investigated cohort. Grammatopoulos et al. [23] reported inferior PROs in hypermobile THA patients with spinal arthrodesis. Our study excluded patients with previous spinal fusion at any level, as this is a known factor influencing the spinopelvic function [4]. Nevertheless, we consider our patient cohort representative for primary THA patients. In line with previous studies, we identified 25% stiff, 60% normal, and 15% hypermobile pelvic mobility [9].

A third reason might be the limited sensitivity of the used valid PRO metrics in terms of detecting differences in this specific biomechanical aspect. In our selected cohort, the used PRO metrics may cluster toward the upper end resulting in a ceiling effect. Even if biomechanics differ, patients may not perceive function differently enough to be detected by these metrics.

Although, no PRO differences between pelvic mobility groups have been detected, our study was able to demonstrate an association of higher pelvic mobility postoperatively with HOOS and HHS as well as functional cup inclination and HOOS 2 years after THA. These findings are in line with the literature underline the connection between cup position and outcomes [10,11]. Innmann et al. [17] also emphasized the importance to address the functional cup orientation. Based on our study, we cannot provide guidance on adapting the cup position. Further studies are needed to elucidate underlying mechanisms influencing spinopelvic biomechanics and outcomes after THA.

Additionally, we would like to emphasize the impact of the complex interplay of multiple trunk, pelvic, and lower extremity muscles on the spinopelvic function. Studies reported an association of increased fat infiltration of the lower back muscles with pelvic retroversion and sagittal spinal malalignment [8,24]. The obstacle is that the musculature influences both the spinopelvic function and the outcome after total hip replacement [25]. Based on the literature, it can be assumed that reduced muscle quality leads to a poorer outcome and also increases the risk of dislocation [26]. As muscle function was not determined radiologically or clinically in our study, no conclusions can be drawn in this regard.

No dislocation event was reported in our cohort. This is expected given the generally low instability rate in primary THA and additionally due to our selection criteria excluding high-risk patients for instability with previous spinal fusion, ankylosing spondylitis, and neurological preexisting conditions.

Some limitations of the study need to be addressed. The FU rate with over 73% is satisfying, nevertheless a selection bias cannot be ruled out. Despite an a priori power analysis and a reached adequate sample size, a larger sample size may have been beneficial to detect differences between the pelvic mobility groups. The exclusion of spinal fusion patients might lead to a selection bias, but is necessary to diminish spinal fusion as an important confounder. We did perform seated radiographs only before THA and at the 1-week FU but not at the 2Y-FU, due to patient safety. We did not perform examinations in deep flexed seated position, which may have limited further insights into spinopelvic function [27].

## Conclusions

Our study could not reveal worse PROs in the impaired pelvic mobility groups in a primary THA cohort at a FU after 2 years. It might be concluded that primary THA patients without risk factors (eg, spinal fusion, neurological disease) do not face limited outcomes, regardless of their preoperative pelvic mobility.

## Data availability

The datasets generated and analyzed during the current study are available from the corresponding author on reasonable request.

## Ethics approval

The institutional review board of Charité-Universitätsmedizin Berlin approved this study under the registration number EA2/021/22.

## CRediT authorship contribution statement

**Henryk Haffer:** Writing – original draft, Visualization, Validation, Supervision, Resources, Project administration, Methodology, Investigation, Funding acquisition, Formal analysis, Data curation, Conceptualization. **Maximilian Muellner:** Writing – review & editing, Validation, Methodology, Data curation, Conceptualization. **Luis Becker:** Writing – review & editing, Validation, Conceptualization. **Abelina Schneider:** Writing – review & editing, Software, Methodology, Formal analysis, Data curation, Conceptualization. **Tom Folkerts:** Writing – review & editing, Validation, Software, Methodology, Data curation. **Sebastian Hardt:** Writing – review & editing, Resources, Investigation. **Carsten Perka:** Writing – review & editing, Resources, Funding acquisition. **Matthias Pumberger:** Writing – review & editing, Validation, Supervision, Resources, Project administration, Methodology, Investigation, Funding acquisition, Conceptualization.

## Conflicts of interest

Carsten Perka receives royalties from Zimmer, Smith & Nephew, and J & J MedTech; is a paid consultant for Zimmer, Smith & Nephew, J & J MedTech, and Link; and serves as a board or committee member for DGOOC, International Hip Society, Arbeitsgemeinschaft Endoprothetik, AO Recon Steering Board. All other authors declare no potential conflicts of interest.

For full disclosure statements refer to https://doi.org/10.1016/j.artd.2026.102148.

## Figures and Tables

**Supplement Table 1:** Measured radiological parameters.

Radiological parameter	Description
Lumbar lordosis (LL)	Angle between superior endplate of L1 and superior endplate of S1.
Pelvic tilt (PT)	Angle between the line joining the midpoint of the hip axis to the midpoint of S1superior endplate and the vertical reference line
Pelvic femoral angle (PFA)	Angle between the center of the hip axis to the midpoint of the superior sacral endplate and a 10-cm line from the center of the hip axis to the ventral cortex of the femur

**Supplement Table 2:** Osteochondrosis intervertebralis was graded according to a Kellgren and Lawrence adjusted score (grades 0 to 4).

Parameter	Quantity/proportion in the cohort
Mild osteochondrosis intervertebralis [N/%]	85/59
Severe osteochondrosis intervertebralis [N/%]	59/41
Sagittal balance (SVA) [N/%]	71/49
Sagittal balance (PI-LL) [N/%]	65/45
Spondylolisthesis (Meyerding 1) [N/%]	19/13
Spondylolisthesis (Meyerding 2) [N/%]	3/2

The highest grade of the classification regarding all levels of the lumbar spine was determined. The patients were then divided into groups: mild-or-severe osteochondrosis intervertebralis. In the mild group, patients were assigned Kellgren and Lawrence grades 0 to 2. Severe osteochondrosis intervertebralis was defined as grades 3 and 4 according to Kellgren and Lawrence. SVA, sagittal vertical axis. SVA >50 mm is defined as imbalance. PI-LL, pelvic incidence minus lumbar lordosis mismatch. PI-LL >10° is defined as imbalance. Spondylolisthesis is graded by the definition of Meyerding. There was no higher grade (Meyerding 3/4) spondylolisthesis in our cohort detected. Spinal Pathology.

## Appendix A Supplementary data

Conflict of Interest Statement for Pumberger

Conflict of Interest Statement for Schneider

Conflict of Interest Statement for Haffer

Conflict of Interest Statement for Perka

Conflict of Interest Statement for Folkerts

Conflict of Interest Statement for Becker

Conflict of Interest Statement for Hardt

Conflict of Interest Statement for Muellner

## References

[1] Bozic K.J., Kurtz S.M., Lau E., Ong K., Vail T.P., Berry D.J. (2009). The epidemiology of revision total hip arthroplasty in the United States. J Bone Joint Surg Am.

[2] Stefl M., Lundergan W., Heckmann N., McKnight B., Ike H., Murgai R. (2017). Spinopelvic mobility and acetabular component position for total hip arthroplasty. Bone Joint J.

[3] Haffer H., Adl Amini D., Perka C., Pumberger M. (2020). The impact of spinopelvic mobility on arthroplasty: implications for hip and spine surgeons. J Clin Med.

[4] An V.V.G., Phan K., Sivakumar B.S., Mobbs R.J., Bruce W.J. (2018). Prior lumbar spinal fusion is associated with an increased risk of dislocation and revision in total hip arthroplasty: a meta-analysis. J Arthroplasty.

[5] Malkani A.L., Garber A.T., Ong K.L., Dimar J.R., Baykal D., Glassman S.D. (2018). Total hip arthroplasty in patients with previous lumbar fusion surgery: are there more dislocations and revisions?. J Arthroplasty.

[6] Esposito C.I., Carroll K.M., Sculco P.K., Padgett D.E., Jerabek S.A., Mayman D.J. (2018). Total hip arthroplasty patients with fixed spinopelvic alignment are at higher risk of hip dislocation. J Arthroplasty.

[7] Ike H., Dorr L.D., Trasolini N., Stefl M., McKnight B., Heckmann N. (2018). Spine-Pelvis-Hip relationship in the functioning of a total hip replacement. J Bone Joint Surg Am.

[8] Muellner M., Haffer H., Chiapparelli E., Dodo Y., Tan E.T., Shue J. (2022). Differences in lumbar paraspinal muscle morphology in patients with sagittal malalignment undergoing posterior lumbar fusion surgery. Eur Spine J.

[9] Innmann M.M., Merle C., Gotterbarm T., Ewerbeck V., Beaule P.E., Grammatopoulos G. (2019). Can spinopelvic mobility be predicted in patients awaiting total hip arthroplasty? A prospective, diagnostic study of patients with end-stage hip osteoarthritis. Bone Joint J.

[10] Polat Y., Aygun Polat E., Alparslan M., Kececi T., Cirakli A. (2025). ABS0648 effect of spinopelvic mobility and functional safe zone alignment on patient-reported outcomes in total hip arthroplasty. Ann Rheum Dis.

[11] Liu J., Criddle S., Gilreath N., Heard J., Daher M., Callanan T. (2025). Impact of spinopelvic parameters on acetabular cup positioning and patient-reported outcomes following total hip arthroplasty. J Orthop.

[12] Haffer H., Hu Z., Wang Z., Müllner M., Hardt S., Pumberger M. (2023). Association of age and spinopelvic function in patients receiving a total hip arthroplasty. Sci Rep.

[13] Lewinnek G.E., Lewis J.L., Tarr R., Compere C.L., Zimmerman J.R. (1978). Dislocations after total hip-replacement arthroplasties. J Bone Joint Surg Am.

[14] Leopold V.J., Homm P.M., Krüger D., Hipfl C., Perka C., Hardt S. (2024). The subjective hip value is a valid, reliable, and responsive instrument for assessing hip function in primary total hip arthroplasty. J Arthroplasty.

[15] Lu M., Zhou Y.X., Du H., Zhang J., Liu J. (2013). Reliability and validity of measuring acetabular component orientation by plain anteroposterior radiographs. Clin Orthop Relat Res.

[16] Haffer H., Wang Z., Hu Z., Hipfl C., Pumberger M. (2021). Acetabular cup position differs in spinopelvic mobility types: a prospective observational study of primary total hip arthroplasty patients. Arch Orthop Trauma Surg.

[17] Innmann M.M., Reichel F., Schaper B., Merle C., Beaulé P.E., Grammatopoulos G. (2021). How does spinopelvic mobility and sagittal functional cup orientation affect patient-reported outcome 1 year after THA?-A prospective diagnostic cohort Study. J Arthroplasty.

[18] Faul F., Erdfelder E., Lang A.G., Buchner A. (2007). G∗Power 3: a flexible statistical power analysis program for the social, behavioral, and biomedical sciences. Behav Res Methods.

[19] Innmann M.M., Verhaegen J.C.F., Reichel F., Schaper B., Merle C., Grammatopoulos G. (2022). Spinopelvic characteristics normalize 1 year after total hip arthroplasty: a prospective, longitudinal, case-controlled Study. J Bone Joint Surg Am.

[20] Ryan N.S., Kowalski E., Antoniades S., Catelli D.S., Beaulé P.E., Lamontagne M. (2024). Do 3-dimensional spinopelvic characteristics normalize after THA? A prospective, comparative Study using motion capture analysis. Clin Orthop Relat Res.

[21] Muellner M., Wang Z., Hu Z., Hardt S., Pumberger M., Becker L. (2022). Hip replacement improves lumbar flexibility and intervertebral disc height - a prospective observational investigation with standing and sitting assessment of patients undergoing total hip arthroplasty. Int Orthop.

[22] Haffer H., Wang Z., Hu Z., Hipfl C., Perka C., Pumberger M. (2022). Total hip replacement influences spinopelvic mobility: a prospective observational Study. J Arthroplasty.

[23] Grammatopoulos G., Gofton W., Jibri Z., Coyle M., Dobransky J., Kreviazuk C. (2019). 2018 Frank Stinchfield award: spinopelvic hypermobility is associated with an Inferior outcome after THA: examining the effect of spinal arthrodesis. Clin Orthop Relat Res.

[24] Muellner M., Haffer H., Moser M., Chiapparelli E., Dodo Y., Amini D.A. (2022). Paraspinal musculature impairment is associated with spinopelvic and spinal malalignment in patients undergoing lumbar fusion surgery. J Spine.

[25] Kawano T., Nankaku M., Murao M., Yuri T., Hamada R., Kitamura G. (2024). Impact of preoperative skeletal muscle quality on functional outcome in total hip arthroplasty. J Am Med Dir Assoc.

[26] Haffer H., Muellner M., Chiapparelli E., Dodo Y., Camino-Willhuber G., Zhu J. (2024). Georg Schmorl Prize of the German Spine Society (DWG) 2023: the influence of sarcopenia and paraspinal muscle composition on patient-reported outcomes: a prospective investigation of lumbar spinal fusion patients with 12-month follow-up. Eur Spine J.

[27] Behery O.A., Vasquez-Montes D., Cizmic Z., Vigdorchik J.M., Buckland A.J. (2020). Can flexed-seated and single-leg standing radiographs be useful in preoperative evaluation of lumbar mobility in total hip arthroplasty?. J Arthroplasty.

