# Supplementary material for: Impaired Preoperative Spinopelvic Function Does Not Compromise the Patient-Reported Outcome at 2-Years After Total Hip Arthroplasty
Source: Arthroplast Today. 2026 Sep 24;41:102148. doi: 10.1016/j.artd.2026.102148 (PMC13634557; doi:10.1016/j.artd.2026.102148)
Supplement: Supplementary file 6 [file mmc6.pdf]

INDIVIDUAL CONFLICT OF INTEREST STATEMENT

American Association of Hip and Knee Surgeons

(Adopted from the American Academy of Orthopaedic Surgeons disclosure statement)

The following form **<u>must be filled out completely and submitted by each author (example, 6 authors, 6 forms).</u>**
<u>All items require a response. If there is no relevant disclosure for a given item, enter "*None*."</u>

Manuscript Title
Impaired Preoperative Spinopelvic Function does not compromise the Patient-Reported Outcome at 2-Years after Total Hip Arthroplasty

1. Royalties from a company or supplier (The following conflicts were disclosed)

None.

2. Speakers bureau/paid presentations for a company or supplier (The following conflicts were disclosed)

None.

3A. Paid employee for a company or supplier (The following conflicts were disclosed)

None.

3B. Paid consultant for a company or supplier (The following conflicts were disclosed)

None.

3C. Unpaid consultants for a company or supplier (The following conflicts were disclosed)

None.

4. Stock or stock options in a company or supplier (The following conflicts were disclosed)

None.

5. Research support from a company or supplier as a Principal Investigator (The following conflicts were disclosed)

None.

6. Other financial or material support from a company or supplier (The following conflicts were disclosed)

None.

7. Royalties, financial or material support from publishers (The following conflicts were disclosed)

None.

8. Medical/Orthopaedic publications editorial/governing board (The following conflicts were disclosed)

None.

9. Board member/committee appointments for a society (The following conflicts were disclosed)

None

Each author must sign AND print or type his/her name, date and submit a separate form

In addition, one BLINDED Conflict of Interest form (no author names used) should be submitted per manuscript with all author disclosures.

Luis Becker	Luis Becker Digital unterschrieben von Luis Becker Datum: 2025.09.24 12:49:59 +02'00'	24.09.2025
Author Name (Print or Type)	Author Signature	Date
